# Hospital Admission Profile Due to Osteoarthritis: An Ecological Study

**DOI:** 10.7759/cureus.38435

**Published:** 2023-05-02

**Authors:** Rakan Ekram, Mai S Nazer

**Affiliations:** 1 Department of Health Information Technology and Management, Faculty of Public Health and Health Informatics, Umm Al-Qura University, Makkah, SAU; 2 Department of Internal Medicine, King Abdullah Medical Complex, Jeddah, SAU

**Keywords:** hospitalisation, wales, osteoarthritis, england, admission

## Abstract

Background

Osteoarthritis (OA) is also known as degenerative joint disease and is considered the major cause of joint pain and disability. Furthermore, OA is the most common, costly, and disabling form of joint diseases. The objective of this study is to explore the hospital admission profile due to OA between the period 1999 and 2019 in England and Wales.

Method

This is an ecological study that used health care data in the United Kingdom. Patients who were hospitalized for OA in England and Wales between 1999 and 2019 formed the study population. The Hospital Episode Statistics in England and the Patient Episode Database for Wales databases were used in this study. The difference in the admission rate during the study period was estimated using the chi-squared test.

Results

The admission rate during the study period increased by 112.1% for all hospital admission related to OA. The most common type of admission was related to gonarthrosis, which accounted for 46.7% of the total number of admissions for OA. The increase in admission rate across different types of admissions related to OA was not consistent. The highest increase in the admission rate was observed for polyarthrosis (604.6%). Admission rates related to OA were observed to be directly related to age. The highest increase in the admission rate during the study period was for the age group of 15-59 years (102.1%). Admission rate due to OA was higher among females compared to males.

Conclusion

The increase in admission rates for the various OA-related admissions was not consistent. This study found that the age range of 15 to 59 years experienced the greatest increase in admission rates. Female gender is a high risk factor for OA, especially in women around menopause.

## Introduction

Osteoarthritis (OA) is a form of arthritis and at the top of joint disorders in the United States, affecting around 25% of adults [1]. OA is a disease where chronic overload and malfunction biomechanics on the joint leads to a destruction of the joint cartilage, resulting in inflammation combined with joint rigidity, swelling, and impaired mobility [2]. Mechanical stress exceeds a joint's capacity to repair and maintain itself, thereby predisposing articular cartilage to premature degeneration. Obesity, high-impact work or sports, and neuromuscular dysfunction are only a few examples of mechanically caused pathophysiological alterations to the articular cartilage, subchondral bone, and other perhaps additional joint tissues. OA is the most common joint disease and a major cause of joint pain and disability [3]. Furthermore, OA is the most common, costly, and disabling form of joint diseases [4]. Multiple factors affect the development and the progress of OA, including age, obesity and weight, and presence of metabolic diseases [5,6].

The leading risk factors that affect the development of OA are ranged between modifiable risk factors and non-modifiable risk factors, gender, race, genetics, past injury or joint damage, and prior ethnicity. Excess weight, certain vocations and activities, joint injury, misaligned joints, and weak quadriceps are among the risk factors for OA that may be controlled [7]. Epidemiological principles are used to describe and understand the prevalence, occurrence, and progression of OA [8]. Examining the prevalence of OA-related admissions enables the decision-makers to identify the trend of the disease itself and whether there is a need for an improvement in health care provision for patients with OA. In addition, it helps in identifying the high-risk population to whom actions should be directed. Meanwhile, regarding symptomatic OA, an estimation for a lifetime risk of having a knee OA is around 14%, ranging from 9.6% for non-obese males to 23.8% for obese females, and higher estimation of the prevalence of symptomatic hand OA accounted for 39.8% [9,10]. Furthermore, according to a previous meta-analysis, the global prevalence of knee OA is 16% [4]. Adults with arthritis in the United States paid $303.5 billion in medical care expenditures in 2013, accounting for 1% of the country's gross domestic product (GDP), and they lost out on $303.5 billion in wages as a result.

OA is a significant ongoing public health problem internationally and can be worse with increased age [11]. OA impacts wide aspects of individual and population health, where hip OA and knee OA are ranked as the 11th highest contributor of global disability and ranked the 38th highest in disability life years as per the Global Burden of Diseases study in 2010 [4]. Meanwhile, OA can affect the quality of life of patients beyond their physical function disability and have an impact on mental health as it causes disability and reduce the quality of life of the patients [12]. Therefore, most patients with OA demand an ongoing care by the community and through solid health care [13]. The intensity of pain and functional impairment may demand hospital admission for hip, hand, and knee OA care management [14]. Therefore, the objective of this study is to explore the trend of hospital admission due to OA between the period 1999 and 2019 in England and Wales. In addition, we aimed to identify the impact of age and gender on this type of admission.

## Materials and methods

Study design

This is an ecological study at the population level that used health care data in the United Kingdom.

Study population

Patients who were hospitalized for OA in England and Wales between 1999 and 2019 were included in the study. There was no restriction on the age, gender, or type of OA.

Data source

The Hospital Episode Statistics in England and the Patient Episode Database for Wales databases were used in this study [15,16]. Population data were extracted from the Office for National Statistics database [17]. Details regarding the quality of these medical databases were described in previous literature [18-21]. These two medical databases were previously used to examine the trend of admissions for various chronic and acute health outcomes. The quality of data reporting in these two medical databases is checked on a regular basis and deemed suitable for research purposes. Data included in these two medical databases cover all health care services provided by the National Health Services. Data in these two medical databases are reported stratified by age (involving four main age groups, which are below 15 years, 15-59 years, 60-74 years, and 75 years and older). Publicly available data do not include information on comorbidities or medication use.

Outcome

We extracted the data for patients from all age groups. Admissions related to OA were identified using the International Classification of Disease (ICD) codes M15-M19.

Statistical analysis

Admission rate was estimated by dividing the number of admissions in any specific year by the mid-year population for the same year. Age-specific admission rates were estimated by dividing the number of admissions for each specific age group by the mid-year population of the same age group in the same year. Gender-specific admission rate was estimated by dividing the number of admissions for males or females by the mid-year population of the same gender in the same year. Admission rates were presented with 95% confidence interval. The difference in the admission rate during the study period was estimated using the chi-squared test. The Statistical Package for Social Science software was used to analyze the data for this study. A two-sided value of p<0.05 was considered statistically significant.

## Results

**Figure 1:**
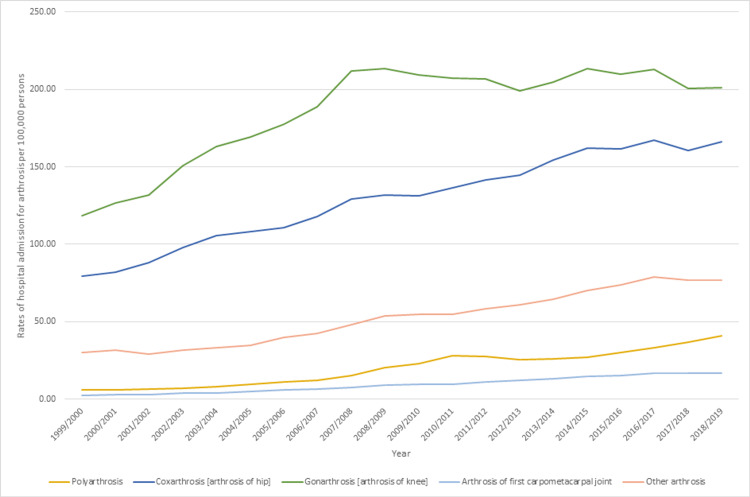
Admission rates for osteoarthritis-related diseases between 1999 and 2019.

The admission rate during the study period increased by 112.1% for all hospital admissions related to OA. There are five main types of admissions that are related to OA: polyarthrosis, coxarthrosis (arthrosis of the hip), gonarthrosis (arthrosis of the knee), arthrosis of the first carpometacarpal joint, and other arthrosis (primary and secondary OA of other joints, shoulders, elbow, wrist, hand, ankle and foot) (Figure 1).

**Figure 2:**
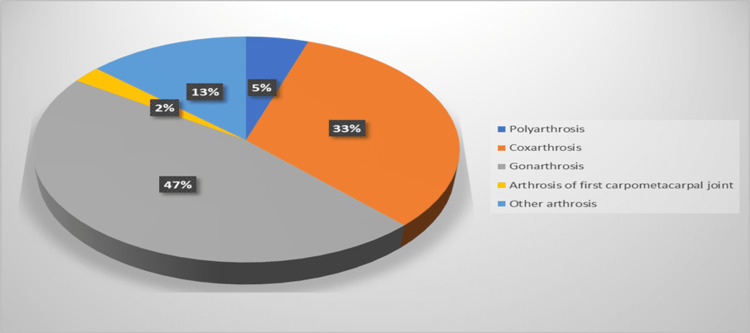
Percentage from the total number of admissions.

The most common type of admission was related to gonarthrosis, which accounted for 46.7% of the total number of admissions for OA (Figure 2).

The increase in admission rate across different types of admissions related to OA was not consistent. The highest increase in the admission rate was observed for polyarthrosis (604.6%), followed by arthrosis of the first carpometacarpal joint (551.5%). Table 1 presents the change in the admission rate across types of admissions that are related to OA.

**Table 1:** Change in admission rates across different types of diseases related to osteoarthritis.

Type of admission	Admission rate in 1999 per 100,000 persons (95% confidence interval)	Admission rate in 2019 per 100,000 persons (95% confidence interval)	Percentage change
All types of osteoarthritis	236.47 (235.15-237.79)	501.64 (499.84-503.43)	112.1%
Polyarthrosis	5.79 (5.58-5.99)	40.78 (40.27-41.30)	604.6%
Coxarthrosis (arthrosis of the hip)	79.59 (78.83-80.36)	166.29 (165.25-167.32)	108.9%
Gonarthrosis (arthrosis of the knee)	118.61 (117.68-119.55)	201.04 (199.90-202.18)	69.5%
Arthrosis of the first carpometacarpal joint	2.55 (2.42-2.69)	16.63 (16.30-16.96)	551.5%
Other arthrosis	29.92 (29.45-30.39)	76.90 (76.19-77.60)	157.0%

Role of age in admission rates

**Figure 3:**
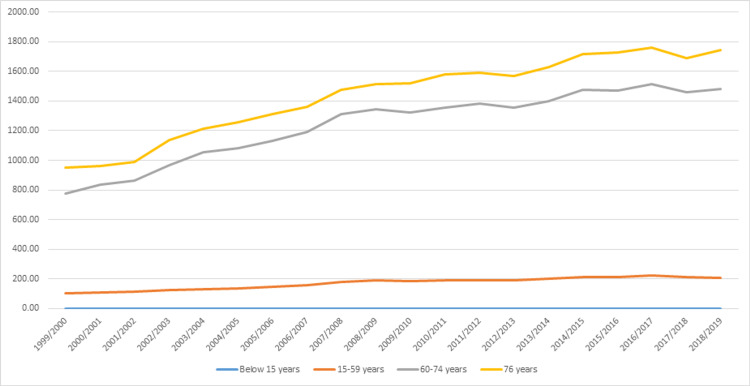
Osteoarthritis admission rates stratified by age.

Admission rates related to OA were observed to be directly related to age (Figure 3). The highest increase in the admission rate during the study period was for the age group of 15-59 years (102.1%), followed by the age group of 60-74 (91.7%) and then the age group of 75 years and above (84.1%) (Table 2).

**Table 2:** Change in admission rates across different age groups.

Age group	Admission rate in 1999 per 100,000 persons (95% confidence interval)	Admission rate in 2019 per 100,000 persons (95% confidence interval)	Percentage change
Below 15 years	0.31 (0.20-0.42)	0.25 (0.16-0.35)	-19.5%
15-59 years	103.37 (102.25-104.50)	208.92 (207.39-210.44)	102.1%
60-74 years	774.06 (767.53-780.58)	1,484.11 (1,476.31-1,491.90)	91.7%
75 years and above	948.45 (938.85-958.05)	1,746.03 (1,734.64-1,757.42)	84.1%

Role of gender on admission rates

**Figure 4:**
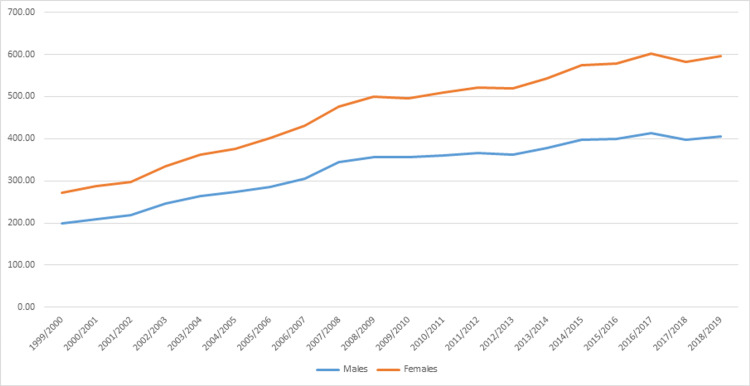
Osteoarthritis admission rates stratified by gender.

Admission rate due to OA was higher among females compared to males (Figure 4). In addition, the increase in the admission rate during the period between 1999 and 2019 was higher among females (118.5%) compared to males (104.2%) (Table 3).

**Table 3:** Change in admission rates stratified by gender

Gender	Admission rate in 1999 per 100,000 persons (95% confidence interval)	Admission rate in 2019 per 100,000 persons (95% confidence interval)	Percentage change
Males	198.49 (196.76-200.22)	405.42 (403.12-407.72)	104.2%
Females	272.64 (270.66-274.62)	595.63 (592.88-598.38)	118.5%

## Discussion

OA is considered the most common joint disorder worldwide [22], with highly increased public health concern [23], which increases the demand for a better understanding of epidemiological factors to reach better health prevention, management, and treatment [24]. Therefore, in this study, we aimed to explore the hospital admission profile due to OA between the period 1999 and 2019 in England and Wales.

Our study found that the admission rate during the study period increased by 112.1% for all hospital admissions related to OA. This increase could be attributed to the increased prevalence of OA in the recent decade [25], which is in line with other studies [4,26], where the increase was reported to be associated with multiple factors including age, obesity, and population expansion, meanwhile the total global population increased by 45% during the period between 1990 and 2019 population as per the United Nation Department of Economic and Social Affairs [27]. Besides, regarding obesity, its presence had tripled between the period 1975 and 2016 [28]; all these factors contributed to the significant increase in hospital admission rates.

There are five main types of admissions in this study that are related to OA, which are “polyarthrosis, coxarthrosis, gonarthrosis, arthrosis of the first carpometacarpal joint, and other arthrosis (primary and secondary OA of other joints, shoulders, elbow, wrist, hand, ankle, and foot)”. Polyarthrosis is defined as the involvement of two or more joints in the joint inflammation, and it needs a close care and monitor, as a delay in the treatment may result in significant morbidity [28]. Meanwhile, coxarthrosis is the arthrosis of the hip with multiple etiologic factors including obesity, malabsorption, and muscle or tendon imbalance [29]. Arthrosis of the first carpometacarpal joint result from joint hypermobility and subluxation [30]. Other arthrosis including primary and secondary OA of other joints, shoulders, elbow, wrist, hand, ankle, and foot.

In our study, gonarthrosis (knee OA) was identified to be the most common type of hospital admission that accounted for 46.7% of the total number of admissions for OA. Indeed, knee OA impacts the quality of life in multiple ways and is also placed at the top of causes of pain and disability worldwide [31]; thus, this may justify this higher incidence and prevalence of knee OA among other OA types that lead to its increased hospital admission rate.

In our study, we found that polyarthritis demonstrated the highest increase in the admission rate and accounted for 604.6%, followed by arthrosis of the first carpometacarpal joint, which accounted for 551.5%. In our study, the increase in admission rate across the different types of admissions related to OA was not consistent; however, the significant increase in polyarthritis admission rates is highly associated with the increased prevalence of polyarthritis [28]. Multiple risk factors contributed to this increased prevalence, including dietary factors that are linked independently to the development of polyarthritis [31]. The increase in hospital admission rate related to the first carpometacarpal joint is associated with the poor and bad quality of lifestyle, which results from the increased and the elevated pain of the joint resulted from the consequences of carpometacarpal arthritis [32].

This study found that the admission rates related to OA were observed to be directly related to age, which is in line with other studies, where the increase in the global lifetime leads to an increase in the prevalence of OA [33]. In addition, age is the main risk factor associated with OA [34]. Our study found that the age group of 15-59 years had the highest increase in the admission rate during the study period and accounted for 102.1%, followed by the age group of 60-74 years with 91.7% increase and then the age group of 75 years and above with 84.1% increase. A previous study on the effect of the age on hip and knee OA found that age is a major risk factor, and it is impact is in a rapid increase between 50 and 75 years of age, while the incident of hand OA peaked in the age of 55 to 60 years, especially in women [35].

Admission rate due to OA was higher among females compared to males, and this increase is due to the effect of female gender as a risk factor for the incidence of OA, especially in women around menopause [35]. Previous literature found that estrogen had a vital role in the development of OA [27]. In addition, in our study, the increase in the admission rate during the period between 1999 and 2019 was higher among females (118.5%) compared to males (104.2%). This is consistent to the findings of a previous study conducted by the Global Burden of Disease between 1990 and 2019, where the prevalence of OA increased with age and was more common among females than males [26].

Future studies are warranted to identify other risk factors that might have an influence on OA-related complications and hospital admissions. In addition, future studies should identify possible effective preventive intervention that might decrease the incidence of OA complications.

Despite the strength point of this study being the first to examine admission profile related to all types of OA among all age groups, this study has limitations. Having data on the population level restricted our ability to identify other important risk factors that might influence admission rate for OA. The data in these two medical databases include readmission episodes, which might lead to overestimation.

## Conclusions

This ecological study found that the rate of admissions due to OA increased markedly in the past two decades. Higher understanding of the epidemiological factors is essential for OA prevention, management, and treatment. Due to its effects on quality of life and the increase in hospital admission rates, OA has become a greater public health concern. This could be achieved by conducting cohort studies at the patient level to identify important risk factors for OA, such as comorbidities, previous surgery, and the use of medications. Numerous causes, including aging, obesity, and population growth, are linked to this trend. Gonarthrosis (knee OA) was the most prevalent type and constituted 46.7% of all admissions. The increase in admission rates for the various OA-related admissions was not consistent. This study found that the age range of 15 to 59 years experienced the greatest increase in admission rates. Female gender is a high risk factor for OA, especially in women around menopause. Future studies should identify effective strategies to decrease the burden of this complication in females.
